# Therapeutic Application of Genome Editing Technologies in Viral Diseases

**DOI:** 10.3390/ijms23105399

**Published:** 2022-05-12

**Authors:** Tae Hyeong Kim, Seong-Wook Lee

**Affiliations:** 1 Department of Molecular Biology, Dankook University, Cheonan 31116, Korea; kth6086@naver.com; 2 Department of Bioconvergence Engineering, Research Institute of Advanced Omics, Dankook University, Yongin 16890, Korea; 3 R&D Center, Rznomics Inc., Seongnam 13486, Korea

**Keywords:** virus, therapeutics, genome editing, ZFN, TALEN, CRISPR/Cas

## Abstract

Viral infections can be fatal and consequently, they are a serious threat to human health. Therefore, the development of vaccines and appropriate antiviral therapeutic agents is essential. Depending on the virus, it can cause an acute or a chronic infection. The characteristics of viruses can act as inhibiting factors for the development of appropriate treatment methods. Genome editing technology, including the use of clustered regularly interspaced short palindromic repeats (CRISPR)-CRISPR-associated (Cas) proteins, zinc-finger nucleases (ZFNs), and transcription activator-like effector nucleases (TALENs), is a technology that can directly target and modify genomic sequences in almost all eukaryotic cells. The development of this technology has greatly expanded its applicability in life science research and gene therapy development. Research on the use of this technology to develop therapeutics for viral diseases is being conducted for various purposes, such as eliminating latent infections or providing resistance to new infections. In this review, we will look at the current status of the development of viral therapeutic agents using genome editing technology and discuss how this technology can be used as a new treatment approach for viral diseases.

## 1. Introduction

Viruses are considered to be on the borderline of living and non-living things, as they display characteristics of both the living and non-living. They are generally composed of genetic materials surrounded by a protein shell. Viruses cannot reproduce by themselves and must infect living organisms and replicate through them. In addition, viruses use the molecular machinery of the infected host cell for multiplication. A new virus is created in the infected host cell through a series of processes and continues to infect other host cells. Consequently, the virus causes acute or chronic disease, depending on how long its host cell remains intact. Some viruses cause simple diseases, such as a cold, while others, such as SARS-Cov-2 (COVID-19), acquired immune deficiency syndrome (AIDS), and hepatitis can cause serious diseases. In addition, some viruses are highly infectious and can cause fatal diseases of the human body, posing a great threat to human health. Hence, the development of effective antiviral treatments is of great interest to the global health community [1].

Genome editing technology involves the use of clustered regularly interspaced short palindromic repeats (CRISPR)-CRISPR-associated (Cas) proteins, zinc-finger nucleases (ZFNs), and transcription activator-like effector nucleases (TALENs) to precisely manipulate specific genomic sequences. This technology allows various manipulations, such as deleting a specific DNA or RNA sequence of the genomic locus or adding a specific sequence to a DNA locus. When genome editing is performed on a target DNA, a nuclease-induced double-stranded break (DSB) generally occurs. Homology-directed repair (HDR) or nonhomologous end-joining (NHEJ) processes then follow to repair the DSB [2]. The development of this technology has enabled sophisticated genomic modifications in various life sciences fields. In particular, it is markedly beneficial in the development of gene therapy.

So far, several genome editing-based antiviral agents have been developed for various purposes, such as eliminating latent infections or providing resistance to new infections [3]. This requires diverse strategies, depending on the characteristics of the target virus and host. In this review, we discuss the progress of genome editing-based antiviral therapeutic agents development and the potential of this method to develop new antiviral therapeutic agents.

## 2. Tools for Genome Editing

**Figure 1:**
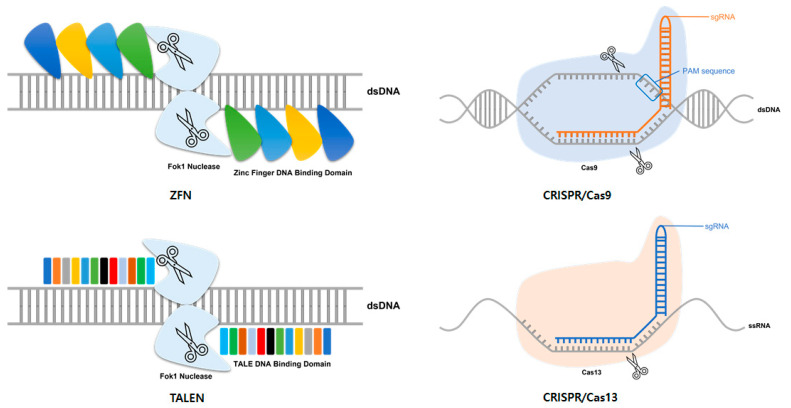
Platforms used for genome editing. Representative tools for genome editing include zinc-finger nucleases (ZFN), transcription activator-like effector nucleases (TALEN), and the clustered regularly interspaced short palindromic repeats (CRISPR)-CRISPR-associated (Cas) system. ZFN is composed of a collection of many zinc-finger DNA binding motifs and a Fok1 endonuclease domain. TALEN is composed of a combination of a DNA binding domain, called TALE, and a Fok1 endonuclease domain. In general, the CRISPR/Cas system is an RNA-guided DNA-cleavage system, such as CRISPR/Cas9. CRISPR/Cas13 is an RNA-guide RNA-cleavage system. Like other CRISPR/Cas systems, it consists of gRNA and Cas protein.

Representative tools for genome editing include ZFN, TALEN, and the CRISPR/Cas system (Table 1), all of which are based on a mechanism that specifically recognizes DNA or RNA existing in nature. ZFNs were originally introduced in the 1990s as the first tools for genome editing [4,5,6]. A general ZFN is composed of a collection of many zinc finger DNA binding motifs and a Fok1 endonuclease domain (Figure 1). Fok1 endonuclease requires dimerization to cleave DNA. Therefore, a pair of ZNFs are required to edit genome-specific sites [7]. Three to six zinc finger motifs are arranged in one ZFN, and one zinc finger motif recognizes three nucleotides. Thus, one ZFN can recognize 9 to 18 nucleotides. When each ZFN is bound to a target site, FoK1 domains become adjacent to each other and are dimerized and activated. The dimerized Fok1 then cleaves DNA. Notably, ZFN played a significant role as a pioneer of genome editing technology. However, it was limited by an off-target effect [8,9,10]. To solve this problem and increase the specificity of ZFNs, various methods have been developed, including utilizing a protein engineering method [11]. However, such methods have a several challenges, such as requiring a lot of time in designing the ZFN and selecting the optimal ZFN; these problems have limited the use of ZFNs in genome editing.

After ZFN, TALEN was designed as a novel genome editing tool [12]. Similar to ZFN, TALEN consists of a repeat sequence-specific DNA-binding domain linked to the Fok1 endonuclease domain, which is a non-specific DNA cleavage domain (Figure 1). This DNA-binding domain consists of a highly conserved repeat sequence derived from a transcription activator-like effector (TALE) derived from a phytopathogenic bacterium *Xanthomonas* [12,13]. Each TALE consists of 33 to 35 amino acids, among which two amino acids, called repeat variable di-residues, determine the specificity for one base pair (bp) [12]. In general, this DNA-binding domain is designed to recognize the DNA of 14–20 bp in length. Like ZFN, TALEN also requires dimerization to activate Fok1 endonuclease. Thus, a pair of TALEN is required for genome editing. Pairs of TALENs are designed to recognize the target site with a space of 12–30 bp. Dimer Fok1 endonuclease causes a DSB in this space. In general, TALEN is known to have high specificity and efficiency, and is easier to design than ZFN [11]. However, to clone a repeat TALE array, a design of a large-scale repeat sequence is required, which limits the development of TALEN. To overcome this obstacle and quickly create a TALE array, a method called Golden Gate cloning has been developed [14,15,16].

The CRISPR locus was first discovered in E. coli in 1987 [17]. Since then, it has been found in several other bacterial species [18]. However, their function was discovered only after similarities with phage DNA sequences were discovered in 2005 [19]. It is known that they attack foreign DNA by inducing RNA-guided DNA cleavage in the adaptive immune response of bacteria and archaea [18,19,20]. The CRISPR/Cas system is divided into two classes: (1) the class I CRISPR/Cas system, which consists of multiprotein effector complexes, and (2) the class II system, which consists of a single effector protein [21,22]. Among these, the class II type 2 CRISPR-Cas system derived from *Streptococcus pyogenes* is the most commonly used system for gene editing. The type 2 CIRSPR/Cas system utilizes Cas9 as an endonuclease. Cas9 is guided to a target by a chimeric CRISPR RNA (crRNA); trans-activating RNA (tracrRNA) also called guide RNA (gRNA) [23]. In genome editing, the chimeric RNA is processed into a single RNA in the form of single-stranded guide RNA (sgRNA) prior to use. The sgRNA contains a sequence of 20 bp that is complementary to the target DNA sequence. Short DNA sequences, called protospacer adjacent motif (PAM), are required at the target site for the target to be recognized and the system to operate. PAM sequences are located right next to the target sequences recognized by the sgRNA. PAM sequences required for Cas9 are known as NGG or NAG [24]. The sgRNA is attached to the target sequence by Watson–Crick base pairing. The induced Cas9 causes DSB (Figure 1) [25,26]. The cleaved DNA is repaired through NHEJ or HDR and used for editing. After the development of genome editing technology using Cas9, various CRISPR/Cas systems have been discovered and developed, including a system using CRISPR/Cas13 [27]. While the general CRISPR/Cas system targets DNA, this system targets RNA without requiring a PAM sequence to operate the system (Figure 1) [28]. The flexibility of this technique offers many advantages for targeting viruses that can mutate rapidly and result in various types of mutations. Consequently, many studies on the utilization of this technology for virus therapy are underway.

## 3. Therapeutic Genome Editing for Double-Stranded DNA Viruses

### 3.1. Therapeutic Genome Editing for Human Papillomavirus (HPV)

Human papillomavirus (HPV) is a non-enveloped double-stranded DNA virus of the family Papilomaviridae. Most HPV infections show no symptoms. Patients can recover within two years. However, in some cases, the infection can cause warts or precancerous lesions, with an increased risk of the cervical, vulval, vaginal, penile, and anal cancers [29]. In particular, HPV is the main cause of cervical cancer, with two strains of HPV (HPV-16 and HPV-18) accounting for 70% of HPV-related cervical cancers. During the viral life cycle, two viral proteins (E6 and E7) are overexpressed to act as oncoproteins. These two proteins can bind to p53 and RB, respectively, to promote degradation and activate the cell cycle of the host cells, increasing the risk of malignant transformation of the infected cells [30]. So far, there are three vaccines (Gardasil, Gardasil 9, and Cervarix). The vaccines have been shown to effectively block the initial infection from a diverse range of HPV types, including HPV-16 and HPV-18 [31]. Other than these vaccines, there is still no effective therapeutic agent for HPV infection or cancer caused by HPV.

#### 3.1.1. Therapeutic Strategies for Targeting HPV Viral Genes

Various groups have developed genome editing-based therapeutic agents for HPV infection or cancer caused by HPV infection, and the development of therapeutic agents that target viral genes or host factors known to interact with viruses is in progress. Most studies have been conducted with the HPV E6/E7 oncogene as a target. Shankar et al. [32] designed a specific TALEN to target the HPV E7 oncogene. They showed that selected TALEN could effectively downregulate the HPV E7 gene at RNA and protein levels, ultimately inducing cell death by necrosis. Hu et al. [33] developed a specific CRISPR/Cas system to target HPV-16 E7 DNA. They showed that the CIRPSR/Cas system could induce apoptosis and growth inhibition of HPV positive cells and effectively downregulate E7 protein to induce the upregulation of pRb, a host tumor suppressor protein. Lao et al. [34] also developed a CRISPR/Cas9 system specifically targeting the HPV E7 oncogene. They showed that Cas9-mediated E7 knockout could significantly inhibit HPV-induced cancerous activity both in vitro and in vivo. Inturi et al. [35] developed a CRISPR/Cas9 system that specifically targets the HPV-18 E6/E7 oncogene. They showed that HPV-18 E7 knockout resulted in decreased E6 expression with activation of the pRb/p21 pathway that could trigger cellular senescence. An important element in the ability of HPV-18 E7 to contribute towards cell transformation is the presence of a Casein Kinase II (CK II) phospho-acceptor site within the CR2 domain of the protein. Basukala et al. [36] developed a specific CRISPR/Cas9 system to target the HPV-18 E7 CKII recognition site. They showed that the E7 CKII phospho-acceptor site played an important role in E7’s activity in cells derived from cervical cancers and suggested that blocking this activity of E7 could have therapeutic potential.

In some studies, additional methods were used to increase the efficiency of genome editing. Fan et al. [37] developed a specific CRISPR/Cas9 system to target the HPV-18 E7 oncogene. They co-transfected CRISPR/Cas9 with 34 nucleotide (nt) non-homologous double-stranded oligodeoxynucleotide (dsODN) and showed that this method improved editing efficiency and reduced off-target effects. Tian et al. [38] developed a specific CRISPR/Cas9 system to target the HPV-18 E6/E7 oncogene. They also developed a gene knockout chain reaction (GKCR) method for continually generating mutagenic disruptions to improve gene knockout efficiency. They showed that the GKCR method produced a significantly higher percentage of insertions or deletions (indels) in HPV-18 E6/E7 oncogenes. It significantly upregulated p53/RB proteins and inhibited the proliferation and motility of HeLa cells.

Studies on safe delivery of the developed system in vitro or in vivo have also been conducted. Jubair et al. [39,40] developed a specific CRISPR/Cas9 system to target HPV E6 and E7 oncogenes. They systemically delivered this system through PEGylated liposomes in vivo and showed that cell death was induced by apoptosis. Yoshiba et al. [41] designed a CRISPR/Cas9 system that can specifically target the HPV E6 oncogene. They expressed this system through the adeno-associated virus (AAV) in HPV positive cell lines and a mouse model and observed that in each model, apoptosis was induced as the expression level of E6 decreased and the expression level of p53 increased. Zhen et al. [42] designed a CRISPR/Cas9 system that can specifically target the HPV-16 E6/E7 oncogene. They delivered it in vitro and in vivo through a long-circulating pH-sensitive cationic nano-liposome complex and showed that the delivered CRISPR/Cas9 system effectively inhibited the proliferation of HPV16-positive cervical cancer cells and induced apoptosis by inactivating the E6/E7 oncogene. Noroozi et al. [43] designed a specific CRIPSR/Cas9 system to target the HPV-18 E6 oncogene. They delivered it in HeLa cells through AAV and showed that AAV-mediated CRISPR/Cas9 delivery could effectively target the HPV E6 gene in HeLa cells, and that HPV E6 gene disruption resulted in a significant increase in p53 protein levels.

#### 3.1.2. Therapeutic Strategies for Targeting HPV-Related Host Factors

There have been several attempts to obtain therapeutic effects by modulating host cellular factors involved in HPV tumorigenesis. Sterile alpha motif and histidine-aspartic domain HD-containing protein 1 (SAMHD1) is a deoxynucleotide triphosphate triphosphohydrolase (dNTPase) enzyme that can regulate intracellular levels of dinucleotide triphosphates (dNTPs) and act as an intrinsic immune response factor [44]. James et al. [45] found that SAMHD1 is transcriptionally regulated by HPV16 and that it can control HPV-16 induced cell proliferation. In addition, the CRISPR/Cas9 removal of SAMHD1 can promote viral replication.

### 3.2. Therapeutic Genome Editing for Herpes Simplex Virus (HSV)

Herpes simplex viruses 1 and 2 (HSV-1 and HSV-2) are linear double-stranded DNA enveloped viruses that belong to the Herpesviridae family. They can infect epithelial tissues and invade the nervous system, where they enter a latent stage of infection. Both viruses persist in the human body by hiding in the cell bodies of neurons. When activated, they can move from neurons to the skin, where virus replication and shedding occur [46]. Most HSV infections do not require treatment. Antiviral therapy is typically only administered when lesions persist for a long time, accompanied by other symptoms and complications. However, to date, no specific HSV treatments have been developed.

Research to develop HSV infection therapeutic agents using genome editing mainly targets the HSV genome. Roehm et al. [47] developed a CRISPR/Cas9 system that specifically targets the HSV-1 genome. Since infected cell protein 0 (ICP0) can activate HSV-1 lytic infection, they showed that CRISPR/Cas9 introduced indel mutations into exon 2 of the ICP0 gene and reduced HSV-1 infectivity in permissive human cell culture models. Further, van Diemen et al. [48] developed a specific CRISPR/Cas9 system that targets the HSV-1 genome for editing multiple genes of HSV-1. They found that HSV-1 replication was effectively abrogated by this system, which successfully limited productive and latent infections of HSV-1. Karpov et al. [49] developed a plasmid-encoded CRISPR/Cas9 system that targets UL52 and UL29 genes of the HSV-1 primase–helicase complex. They showed that it completely suppressed HSV-1 infection in vero cells within two days. Chen et al. [50] developed a CRISPR/Cas9 system that specifically targets the ICP0 and ICP4 genes of HSV-1 and found that it could effectively inhibit the proliferation of HSV-1 without affecting cell viability. Khodadad et al. [51] designed several sgRNAs targeting HSV-1 viral genes and found that a significant reduction in HSV-1 infection was achieved using the CRISPR/Cas9 system specifically targeting the gD gene of HSV-1.

Another strategy is to target cellular factors related to HSV-1 infection. Li et al. [52] developed a CRISPR/Cas9 system targeting human nectin cell adhesion molecule 1 (NECTIN-1), the major HSV receptor on human corneal epithelial cells (HCECs). They showed that the HSV infection rate of HCECs in knockdown groups was dramatically decreased after the editing of NECTIC-1.

### 3.3. Therapeutic Genome Editing for Epstein–Barr Virus (EBV)

Epstein–Barr Virus (EBV) is a linear double-stranded DNA enveloped virus that belong to the Herpesviridae family. EBV infects B cells and epithelial cells of the immune system. After the initial lytic infection is properly controlled by the immune system, it remains dormant in B cells for life [53]. EBV is the leading cause of infectious mononucleosis. Most EBV infections obtain acquired immunity before serious symptoms develop. However, they can lead to nonmalignant precancerous or malignant EBV-associated lymphoproliferative diseases, such as Burkitt’s lymphoma, hemophagocytic lymphohistiocytosis, and Hodgkin’s lymphoma [54]. No effective treatments for EBV infection have been developed.

Various groups have developed genome editing-based therapeutic agents for EBV infection. Yuen et al. [55] developed a CRISPR/Cas9 system targeting the EBV genome. They used two gRNAs that directly target the promoter region of BamHI-A rightward transcript (BART), which encodes viral microRNAs (miRNAs). Targeted editing was achieved in several human epithelial cell lines latently infected with EBV, and the CRISPR/Cas9-mediated editing of the EBV genome was efficient.

Another strategy is to target cellular factors related to EBV infection. EBV infection and nasopharyngeal carcinoma (NPC) have been widely recognized in recent decades [56]. In particular, latent membrane protein 1 (LMP1) is known to be a factor related to the carcinogenesis of EBV [57]. Huo et al. [58] developed a CRISPR/Cas9 system targeting the LMP1 gene. They confirmed the LMP1-mediated promotion of NPC cell growth, and such promotion can be effectively blocked by the CRISPR/Cas9-mediated LMP1 knockout.

## 4. Therapeutic Genome Editing for Positive Sense Single-Stranded RNA Viruses

### 4.1. Therapeutic Genome Editing for Hepatitis C Virus (HCV)

HCV is a small, enveloped virus of the family Flaviviridae. It has a positive-sense single-stranded RNA as a genome. This genome consists of an untranslated (UTR) region (at 5′ and 3′ ends) and a region coding for a viral polyprotein. HCV causes hepatitis C, liver fibrosis, cirrhosis, and liver cancer [59]. Thus, HCV infection is a major global public health concern. Specifically, the high variability of HCV helps it to evade the host’s immune response, leading to a poor prognosis, even with an anti-HCV treatment. Currently, there are no effective vaccines for HCV. Fortunately, newly developed treatments using pan-genotypic direct-acting antivirals (DAAs) have a short duration (12~24 weeks), with few side effects and a 90% cure rate, regardless of HCV genotype [60]. However, these treatments are too expensive. Moreover, they are susceptible to new resistant viruses. Therefore, the development of more effective new treatments is necessary.

As with other general virus-targeting genome editing technologies, the development of HCV therapeutic agents through genome editing also targets the HCV genome or host cellular factors related to HCV infection. Clement et al. [61] developed a specific CRISPR/Cas9 system that targets Claudin-1 (CLDN1), a major HCV receptor in the hepatocellular carcinoma Huh7.5.1 monoclonal cell line. They showed that the engineered cell line was resistant to HCV infection. Ashraf et al. [62] developed a CRISPR/Cas system that specifically targets the HCV internal-ribosome entry site (IRES) using Cas13a. It has been shown that the Cas13a enzyme can target ssRNA viruses effectively [63]. Similarly, they found that the CRISPR/Cas system they developed significantly inhibited HCV replication and translation in huh-7.5 cells, with minimal effects on cell viability.

### 4.2. Therapeutic Genome Editing for Zika Virus (ZIKV)

Zika virus (ZIKV) is a single-stranded positive-sense RNA enveloped virus of the family Flaviviridae. The ZIKV genome is directly translated into a viral polyprotein that encodes three structural proteins and seven non-structural proteins. The ZIKV RNA genome’s replication depends on the synthesis of double-stranded RNA from its single-stranded positive-sense RNA genome, after which the replication of new single-stranded positive-sense RNA proceeds [64]. ZIKV causes Zika fever, which typically has no symptoms or only mild symptoms. However, when ZIKV infects pregnant women, it can also infect the developing fetus, potentially causing microcephaly, severe brain malformations, and other birth defects. Additionally, in rare cases, adult infection can lead to Guillain–Barré syndrome [65]. To data, there are no effective vaccines or treatments for ZIKV, although several groups have developed ZIKV therapeutic agents using genome editing tools.

The family of adenosine deaminases acting on dsRNA (ADARs) is a human host factor important for the genetic diversity and evolution of ZIKV. Zhou et al. [66] developed a CRISPR/Cas9-based gene editing system to knockout ADAR1. ADAR1 knockout significantly reduced ZIKV RNA synthesis, protein levels, and viral titers in several human cell lines. The expression of the ankyrin repeat and sterile motif domain containing 4b (ANKS4B) in cultured cells and neonatal mice is downregulated by ZIKV infection [67]. Lin et al. [68] generated two ANKS4B knockout (KO) cell clones in A549 and Huh7 cells through the CRISPR/Cas9 gene editing system. In ANKS4B-KO cells, viral replication levels were significantly enhanced with inhibition of autophagy. This reveals that ANKS4B is a new target for ZIKV therapeutics.

### 4.3. Therapeutic Genome Editing for Coronavirus (CoV)

Coronavirus (CoV) is a positive-sense single-stranded RNA virus of the family Coronaviridae, with a genome of about 30 kilobases in size. CoV’s genome is organized as 5′-leader-UTR-replicase (ORF1ab)—spike (S)—envelope (E)—membrane (M)—nucleocapsid (N)—3′UTR-poly (A)—tail. Thus, it can act like a messenger RNA (mRNA) [69]. CoV releases its genome into host cells, where it can be directly translated by the host cell’s ribosomes to create two large overlapping polyproteins. These viral polyproteins are processed to each viral protein that has an essential role in the viral life cycle. CoV mainly infects the respiratory tract, causing mild to fatal disease. In particular, CoV is drawing attention as the cause of the largest pandemic of the 21st century.

In 2003, severe acute respiratory syndrome (SARS) caused by severe acute respiratory syndrome coronavirus (SARS-CoV) began in Asia and spread rapidly. So far, SARS-CoV has infected more than 8000 people, killing approximately 10% of them [70]. In September 2012, a new type of CoV was identified. It was named Middle East respiratory syndrome coronavirus (MERS-CoV). Up to December 2019, 2468 people were infected with MERS-CoV with a mortality rate of 34.5%. In December 2019, a type of pneumonia that was caused by CoV infection was reported in Wuhan, China. This virus was named SARS-Cov-2. It is the virus that causes Coronavirus disease 2019 (COVID-19). SARS-Cov-2 was identified to share about 70% genetic similarities with SARS-CoV [71]. By 28 March 2022, more than 400 million people had been infected and millions had died. Currently, vaccines based on various platforms such as mRNA, DNA, recombinant protein, and viruses have been developed and inoculation is in progress. However, the frequent occurrence of SARS-Cov-2 mutants, such as Delta and Omicron necessitates, the development of vaccines and therapeutics with excellent performance across a wide range of genotypes. Although this pandemic is still ongoing, currently there is no effective treatment that works broadly for COVID-19.

CRISPR-Cas13 is an RNA-guided RNA-targeting CRISPR system. The characteristic of this system may be used to target the RNA of SARS-CoV-2 and inhibit its replication. Abbott et al. [72] developed a CRISPR/Cas13-based strategy, called prophylactic antiviral CRISPR in human cells (PAC-MAN), for viral inhibition that can effectively degrade RNA from SARS-CoV-2 sequences. They designed and screened CRISPR RNAs (crRNAs) targeting conserved viral regions and identified functional crRNAs targeting SARS-CoV-2. This bioinformatic analysis showed that a group of only six crRNAs could target more than 90% of all the coronavirus types. Blanchard et al. [73] also developed a CRISPR/Cas13-based system targeting SARS-CoV-2. They designed crRNAs specific for replicase and nucleocapsid genes of SARS-CoV-2 and showed that selected crRNAs and Cas13a delivery reduced SARS-CoV-2 replication and reduced symptoms in hamsters. Another strategy is to use a Type III CRISPR-based RNA editing system. Lin et al. [74] developed a type III CRISPR-based RNA editing system against SARS-Cov-2, called type III CRISPR endonuclease antivirals for coronaviruses (TEAR-CoV). They showed that the TEAR-CoV-based RNA engineering approach could lead to RNA-guided transcript degradation, both in vitro and in eukaryotic cells.

## 5. Therapeutic Genome Editing for Negative Sense Single-Stranded RNA Viruses

### Therapeutic Genome Editing for Influenza Virus (IV)

The influenza virus is an enveloped virus with a segmented negative single-stranded RNA as its genome. It is a member of the family Orthomyxoviridae. There are four types of this virus: type A, type B, type C, and type D. However, only types A and B are clinically relevant to humans [75]. The influenza virus has eight single-stranded RNA fragments as its genome. Each fragment has one or two genes. A group of 8 RNA fragments encode a total of 15 viral proteins. Due to its high infectivity and variability, the influenza virus has caused serious pandemics several times during the 20th and 21st centuries. In 1918, the Spanish influenza virus killed 20–50 million people. In 1957, the Asian influenza virus recorded approximately 1.1 million deaths. In 1968, the Hong Kong influenza virus caused 1 million deaths worldwide [76,77]. In 2009, a new influenza A virus subtype H1N1 (pH1N1) emerged. Due to its high infectivity, it rapidly spread worldwide and caused the first pandemic in the 21st century [78,79]. In addition, it has a high mutation-dependent diversity. Therefore, the development of an appropriate treatment is required.

It seems that the CRISPR/Cas13-based genome editing system can play a role in the development of influenza virus treatment, similar to other RNA viruses. Abbott et al. [72] developed a CRISPR/Cas13-based strategy, called prophylactic antiviral CRISPR in human cells (PAC-MAN), for viral inhibition that can effectively degrade RNA from the live influenza A virus (IAV). They showed that crRNAs targeting IAV effectively reduced the H1N1 IAV load in respiratory epithelial cells. Blanchard et al. [73] also developed a CRISPR/Cas13-based system for treating the influenza virus. They designed crRNAs specific for PB1 and highly conserved regions of the PB2 of influenza virus and showed that selected crRNAs and Cas13a protein could reduce viral RNA levels efficiently in cell cultures. In addition, Cas13a efficiently degraded influenza RNA in the lung tissues of mice.

## 6. Therapeutic Genome Editing for Single-Stranded RNA Viruses with DNA Intermediate

### 6.1. Therapeutic Genome Editing for Human Immunodeficiency Virus (HIV)

HIV is a lentivirus (a subgroup of retroviruses) that causes acquired immunodeficiency syndrome (AIDS) [80]. It is an enveloped virus with two copies of positive-sense single-stranded RNA as its genome. It can infect human immune cells such as CD4+ T cells, macrophages, and dendritic cells. The first step of viral entry involves the attachment of CD4 binding domains of viral gp120 to CD4 [81]. In this step, HIV uses C–C chemokine receptor type 5 (CCR5) or C–X–C chemokine receptor type 4 (CXCR4) as a co-receptor to invade the target cells. After attachment with these receptors, fusion occurs between cellular membranes and the viral envelope. Viral components are then injected into the host cell. An enzyme called reverse transcriptase releases the positive-sense single-stranded RNA genome from the attached viral proteins and copies it into a complementary DNA (cDNA) molecule [82]. The process of reverse transcription is extremely error-prone. Therefore, numerous mutations occur in the produced HIV genomes, allowing the virus to effectively evade the host’s immune system. After replication of the viral genome, the cDNA and its complement form of a double-stranded viral DNA (provirus) are integrated into the target cell’s chromosome. The integrated DNA provirus is transcribed into RNA to produce mature mRNAs. These mRNAs are then translated into regulatory proteins Tat and Rev. The provirus’s integration into the host genome can lead to either a chronic or acute infection. As a result, new viral particles are created, and host cells are destroyed.

There is no complete cure for HIV/AIDS yet. However, current treatments, the antiretroviral therapies, slow the progression of the disease and reduce the risk of death. They include a combination of three or more antiretroviral drugs (cART) from several different drug classes. Unfortunately, cART has numerous shortcomings, such as severe side effects and high cost. Therefore, genome editing-based systems might be a potential therapeutic approach to overcome the disadvantages of conventional HIV treatment.

#### 6.1.1. ZFN-Based Therapeutic Strategies for Targeting HIV

The development of HIV therapeutic agents based on genome editing technology has mainly targeted the HIV genome, provirus, or related host factors. In particular, many studies have been conducted targeting CCR5 and CXCR4, which are co-receptors for HIV entry. Perez et al. [83] generated ZFN to disrupt endogenous CCR5. They showed that ZFN specifically disrupted ~50% of CCR5 alleles in a pool of primary human CD4+ T cells, and that stable and heritable protection occurred against HIV-1 infection in vitro and in vivo in a NOG model of HIV infection. Tebas et al. [84,85] edited CCR5 in autologous CD4 T cells of persons infected with HIV. They found that HIV RNA became undetectable in one of four patients who could be evaluated, and that blood levels of HIV DNA were decreased in most patients. Yi et al. [86] also edited the CCR5 gene of CD4+ T cells by transient ZFN expression using a nonintegrating lentivirus (NILV) and found that such cells are resistant to HIV-1 infection, both in vitro and in vivo. Wang et al. [87] edited human CXCR4 in CD4+ T cells using ZFN. After engrafting the modified cells into mice, they found lower viral levels in these mice than in mice engrafted with unmodified CD4+ T cells.

Targeting the HIV genome, such as the HIV proviral DNA, is another strategy for developing HIV therapeutic agents. Qu et al. [88] and Ji et al. [89] directly mediated the deletion of the integrated full-length HIV provirus from infected and latently infected human T cell genomes using specially designed ZFN to target a sequence within the LTR. They observed that the frequency of excision was 45.9% in infected human cell lines after treatment with ZFN-LTR, without significant host-cell genotoxicity, suggesting that this strategy could offer a novel approach for eradicating the HIV-1 virus from the infected host in the future.

#### 6.1.2. TALEN-Based Therapeutic Strategies for Targeting HIV

Research on the development of HIV treatment based on TALEN has also been conducted in several studies. Shi et al. [90] designed TALENs targeting multiple regions of the CCR5 gene (CCR5-TALEN). They showed that these CCR5-TALENs were highly functional nucleases that produced protective genetic alterations in human CCR5. In addition, the application of these TALENs directly to primary CD4+ T cells and CD34+ hematopoietic stem cells (HSCs) of infected individuals could create an immune system resistant to HIV-1 infection. The CCR5 Δ32/Δ32 genotype may cure patients infected with HIV-1. Yu et al. [91] used TALEN to reproduce the homozygous CCR5 Δ32 mutation in CD4+ U87 cells. In their study, the HIV-1 challenge test demonstrated that CCR5Δ32/Δ32 CD4+ U87 cells were resistant to HIV infection. Romito et al. [92] also designed TALEN to disturb the CCR5 gene. When edited T cells were challenged with CCR5-tropic HIV, protection in a dose-dependent manner was observed.

Studies targeting the HIV genome have also been conducted. Ebina et al. [93] designed TALEN targeting HIV long terminal repeats (LTR) for excision of HIV proviral DNA. They showed that more than 80% of the DNA was successfully removed from T cell lines. Strong et al. [94] designed TALEN targeting the transactivation response element (TAR) of the HIV-1 proviral DNA. Selected TALEN cleaved proviral DNA in vitro and the full-length integrated proviral DNA genome in living cells. They demonstrated that damaging integrated HIV proviral DNA might be a potential approach for HIV-1 proviral DNA eradication.

#### 6.1.3. CRISPR/Cas-Based Therapeutic Strategies for Targeting HIV

##### CRISPR/Cas System for Targeting HIV Co-Receptor CCR5 and CXCR4

The genome editing technology based on CRISPR/Cas is one of the most popular platforms for HIV treatment development. Like ZFN and TALEN, the CRISPR/Cas system has also been studied for co-receptors such as CCR5 and CXCR4. Wang et al. [95] developed a CRISPR/Cas9 system against the CCR5 gene using a lentiviral vector. This system effectively disrupted the CCR5 gene. They found that the CCR5 gene-disrupted cells were resistant to HIV-1 infection. Xu et al. [96] established a CRISPR/Cas9 system in human CD34+ hematopoietic stem/ progenitor cells (HSPCs) and achieved efficient CCR5 disruption in a mouse model. HIV-1 resistance was observed in the mouse model, as indicated by a significant reduction in virus titration and enrichment of human CD4+ T cells. Xiao et al. [97] also edited the CCR5 gene in human primary CD4+ T cell using the CRIPSR/Cas9 system. Selected sgRNA effectively disrupted the CCR5 gene, and the CCR5-disrupted CD4+ T cells showed increased resistance against HIV-1 infection. Moreover, humanized mice engrafted with CCR5-disrupted CD4+ T cells showed selective survival and enrichment when they were challenged with HIV-1. Liu et al. [98] used a CRISPR/AsCpf1 system to disrupt the CCR5 gene in the human CD4+ T cells. The CRISPR/Cpf1 system has many advantages over the CRISPR/Cas9, such as lower off-target effects, a smaller nuclease size, and a better sgRNA design for multiplex gene editing. They showed that edited cells resisted R5-tropic HIV-1 infection, but not X4-tropic HIV-1 infection, better than the control group in different cell types.

In addition to targeting CCR5, studies targeting CXCR4 or both have also been conducted. Wang et al. [99] designed a CRISPR/Cas9 system targeting CXCR4. Selected sgRNA efficiently induced editing of the CXCR4 gene in human CD4+ cell lines and made these cell lines resistant to HIV-1 infection. Liu et al. [100] used CRISPR-Cas9, combined with piggyBac transposon technologies, to efficiently induce CXCR4 disruption in an HIV-1 reporter cell line. They showed a decline in HIV-1 replication in biallelic CXCR4 gene-edited cells. Liu et al. [101] designed a CRISPR/Cas9 system for HIV-1 treatment using two different gRNA combinations, targeting both CXCR4 and CCR5. This system successfully induced CXCR4 and CCR5 genes editing in various cell lines and primary CD4+ T cells. They showed that CXCR4-tropic or CCR5-tropic HIV-1 infections were significantly reduced in CXCR4- and CCR5-modified cells. Yu et al. [102] ablated CCR5 and CXCR4 genes in human CD4+ cell lines and primary CD4+ T cells simultaneously, using the CRISPR/Cas9 system to efficiently modify both genes in each cell line. These modified cell lines showed resistance to HIV-1 infection.

##### CRISPR/Cas System for Targeting HIV Genome

HIV remains incurable due to the permanent integration of the viral genome to the host chromosome, which increases the risk of viral reactivation even after antiviral therapy. Therefore, another way to use the CRISPR/Cas system for the treatment of HIV is to target the genome, such as the HIV provirus. Hu et al. [103] used the CRISPR/Cas9 system to eliminate the integrated HIV-1 genome by targeting the HIV-1 LTR U3 region. This system completely excised a 9709-bp fragment of integrated proviral DNA. They found that CRISPR/Cas9 expressing cells prevented HIV-1 infection. Zhu et al. [104] tested 10 sites in HIV-1 DNA that can be targeted by CRISPR/Cas9. Sequencing results showed that each target site in HIV-1 DNA was efficiently mutated by CRISPR/Cas9, with the target site in the second exon of Rev (called T10) exhibiting the highest degree of mutation. As a result, HIV-1 gene expression and virus production were significantly diminished, with T10 causing a 20-fold reduction. Kaminski et al. [105] designed a CRISPR/Cas9 DNA editing system to precisely remove the entire HIV-1 genome spanning between 5′ and 3′ LTRs of integrated HIV-1 proviral DNA copies from latently infected human CD4+ T cells. In particular, lentivirus-delivered CRISPR/Cas9 significantly diminished HIV-1 replication in infected primary CD4+ T-cell cultures and drastically reduced the viral load in ex vivo culture of CD4+ T cells obtained from HIV-1 infected patients. Ophinni et al. [106] designed a CRISPR/Cas9 system targeting HIV-1 regulatory genes tat and rev. When CRISPR/Cas9 was tested in persistently and latently HIV-1-infected T-cell lines, inhibition of viral replication in infected T-cell cultures was observed. Chung et al. [107] tested several sgRNAs targeting the HIV-1 LTR region. Among them, a sgRNA targeting nuclear factor kB (NF-kB) binding sites showed no detectable CRISPR-induced off-target edits in HeLa cells. However, 5′ LTR-driven HIV-1 transcription was significantly reduced in three HIV-1 reporter cell lines.

However, this system is associated with viral escape caused by the off-target effect. Consequently, several groups have tried to use various sgRNAs to target various regions of the genome. Wang et al. [108] developed a CRISPR/Cas9 system targeting HIV-1 proviral DNA. They used each set of two gRNAs to effectively edit DNA. They demonstrated that combinations of two antiviral gRNAs delayed viral escape, and identified two gRNA combinations that could durably block virus replication. Lebbink et al. [109] also used a combinatorial approach of two strong gRNAs targeting different regions of the HIV genome. They showed that this system completely abrogated viral replication and prevented viral escape. Yin et al. [110] tested the efficacies of multiplex sgRNAs in several animal models for excision of HIV-1 provirus and observed excision of HIV-1 proviral DNA in several mouse models via AAV delivery. Zhao et al. [111] used combinatorial possibilities for a therapy based on the CRISPR-Cas9 and RNA interference (RNAi) mechanisms that attack viral DNA and RNA. When two different sites in the HIV-1 genome were targeted, either with dual CRISPR-Cas9 antivirals or with a combination of CRISPR-Cas9 and RNAi antivirals, they observed additive inhibition.

Directly targeting the HIV RNA genome is another approach for developing therapeutic agents for HIV. Since the CRISPR/Cas13 system, an RNA-guided RNA cleavage system, can target ssRNA, it can be a powerful tool for developing therapeutic agents for viruses that have ssRNA as their genome. Nguyen et al. [112] designed a CRISPR/Cas13d system targeting highly conserved regions of HIV-1. They showed that the combination with HIV-specific gRNAs efficiently inhibited HIV-1 replication in cell line models. They also showed an effective HIV-1 inhibition in primary CD4+ T cells and the suppression of HIV-1 reactivated from latently infected cells.

## 7. Therapeutic Genome Editing for Single-Stranded DNA Viruses with RNA Intermediate

### Therapeutic Genome Editing for Hepatitis B Virus (HBV)

Hepatitis B virus (HBV) is a partially double-stranded DNA virus that belongs to the Hepadnaviridae family. It is classified into eight genotypes from A to H. The HBV viral DNA genome is rendered to fully double-stranded DNA by cellular DNA polymerases and transcribed by cellular RNA polymerases. Transcribed viral RNAs are translated into viral proteins essential for the virus’s life cycle. HBV causes hepatitis B, which can be either acute or chronic, and may lead to liver cirrhosis and hepatocellular carcinoma. Despite an effective HBV vaccine, it is still a global health concern due to the lack of adequate treatments.

The virus persists in infected hepatocytes, because covalently closed circular DNA (cccDNA), the template for the transcription of viral RNAs, is stable in nondividing cells. Major treatments for HBV infection include the use of interferon-α and nucleotide analogs. However, they cannot eradicate cccDNA. Therefore, many studies targeting HBV cccDNA have been conducted. Dong et al. [113] designed a CRISPR/Cas9 system that targets conserved regions of the HBV genome. This system reduced viral production. In addition, CRISPR/Cas9-directed cleavage and cleavage-mediated mutagenesis occurred in the HBV cccDNA of transfected cells. In a mouse model, the injection of sgRNA–Cas9 resulted in low levels of cccDNA and HBV protein. Kennedy et al. [114] also designed a CRISPR/Cas9 system targeting HBV cccDNA and observed the effective inhibition of HBV DNA production in in vitro models of both chronic and de novo HBV infection. Liu et al. [115] designed eight gRNAs that target conserved regions of different HBV genotypes to disrupt HBV cccDNA and found that this HBV-specific CRISPR/Cas9 system could inhibit the replication of HBV and decrease viral DNA significantly, both in vitro and in vivo. Stone et al. [116] developed an AAV vector to edit the HBV genome in liver-humanized FRG mice chronically infected with HBV through the CRISPR/Cas9 system and found that the HBV-specific AAV-Cas9 therapy significantly improved the human hepatocytes survival and showed a trend toward decreasing the amount of total liver HBV DNA and cccDNA. Yan et al. [117] also used the CRISPR/Cas9 system to disrupt HBV cccDNA via the AAV vector using a liver-specific promoter for the specific expression of Cas9 in the liver and found that it reduced the level of HBV infection in a cell culture system.

The off-target effect is one of the biggest challenges in the development of HBV treatment. Therefore, there have been efforts to develop therapeutic agents to prevent such off-target effects. Induction of a double-strand break (DSB) on the targeted genome by Cas9 risks an unwanted off-target effect on the host genome. Cas9-nickase can cleave a single strand of DNA. Therefore, two sgRNAs are required to induce DSBs using the Cas9-nickase, lessening the unwanted off-target effects. Kurihara et al. [118] examined the effects of expressing Cas9-nickase and nuclease dead Cas9 (dCas9) with sgRNAs on HBV replication. Cas9-nickase expression with two sgRNAs cleaved the target HBV genome and suppressed the viral-protein expression and HBV replication, both in vitro and in vivo. In addition, dCas9 expression with sgRNAs suppressed HBV replication in vitro, without cleaving the HBV genome. Yang et al. [119] examined the utility of a recently developed CRISPR/Cas-mediated “base editors” (BEs) for inactivating HBV gene expression without the cleavage of DNA. Cas9-BE with certain gRNAs effectively base-edited polymerase and surface genes and reduced HBV gene expression in cells harboring integrated HBV genomes, with very few indels induced.

## 8. Therapeutic Genome Editing in Clinical Trials for Viral Diseases

The genome editing technology is a relatively recently developed technology. However, the convenience and potential of this technology have made great strides in many fields of life science. Efforts have been made to use this technology to develop therapeutic agents for viral diseases, and several groups have used the technology in clinical practice.

Perez et al. [83] generated ZFN which can disrupt 50% of CCR5 alleles in a pool of primary human CD4+ T cells. They found that HIV-1-infected mice engrafted with the ZFN-modified CD4+ T cells had lower viral loads and higher CD4+ T-cell counts than mice engrafted with wild-type CD4+ T cells. The same group conducted clinical studies of the HIV treatment based on previous preclinical studies [84,85]. An open-label, nonrandomized, uncontrolled study of a single dose of ZFN-modified autologous CD4 T cells showed HIV RNA undetectable in one of four HIV patients and a decrease in the blood level of HIV DNA in most patients, with safety [84]. Moreover, a phase I clinical trial that infused CCR5 gene-edited CD4+ T cells showed that infusion of the CD4+ T cells was well tolerated, with no serious adverse events [85]. They observed a modest delay in the time to viral rebound relative to controls in the phase I study. However, 3 of the 14 patients, two of whom were heterozygous for CCR Δ32, showed post-viral rebound control of viremia, with substantial restoration of HIV-specific CD8+ T cell responses, demonstrating that the CCR5 gene-edited CD4+ T cell infusion could aid in the cure of HIV through augmenting pre-existing HIV-specific immune responses.

Excision Bio Therapeutics recently received U.S. Food and Drug Administration approval for the clinical trial of EBT-101 for HIV-1-infected patients (https://clinicaltrials.gov/ct2/show/NCT05144386, 15 September 2021). EBT-101 is an AAV9-based CRISPR/Cas9 system targeting the proviral DNA of HIV-1 and composed of Cas9 and three gRNAs. Many other clinical studies based on genome editing for antiviral therapy are expected to proceed in the near future.

## 9. Conclusions

**Figure 2:**
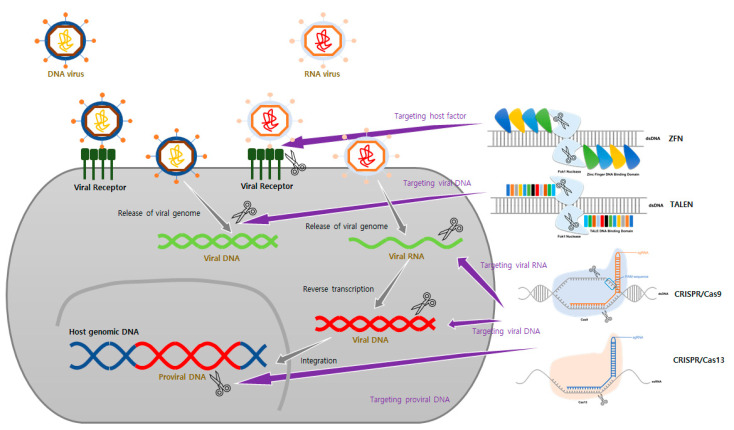
Schematic of genome editing tools used to develop antiviral therapeutic agents. According to each stage of the virus life cycle, targets of genome editing tools can vary. (1) It suppresses the life cycle of a virus by targeting host factors that interact at various stages, such as entry and replication of the virus. (2) Virus replication can be inhibited by directly targeting the genome of a virus that has DNA or RNA as its genome. (3) It is also possible to develop therapeutic agents by targeting the provirus, the genome of a virus that has been integrated into the host genome.

Over the past 10 years, there have many studies on specific genome editing technologies-based therapies (Table 2, Figure 2). It has become possible to quickly and easily change the sequence of genetic information through genome editing technology. The advantages of genome editing technologies have enabled many studies on viral diseases that otherwise would have been difficult to conduct using older methods. In particular, the flexibility of the CRISPR/Cas system technology has made it possible to deal with diseases caused by substances such as highly mutated viruses. For this reason, this technology has made rapid progress over the past decade. However, there are challenges in applying this technology as an actual treatment, and many research studies are aimed at finding solutions to these problems.

First, unwanted mutations in genomic loci, called off-target effects, can potentially cause genomic toxicity, genome instability, disruption of gene function, epigenetic alterations, and even carcinogenesis, which can be fatal [120,121]. Therefore, increasing the efficiency and accuracy of gene correction using genome editing tools remains a major task. In particular, DSBs in unwanted genomic loci can be fatal. Therefore, instead of the DSBs approach, attempts have been made to induce more sophisticated correction by utilizing nickase [118]. This strategy can significantly avoid off-target cleavage without reducing the efficiency of genome editing. When using the CRISPR/Cas system, both the structure and composition of gRNA can affect the level of off-target effects [122]. The use of truncated and less-active sgRNAs that are shortened at the 5′ end by two to three nucleotides can decrease undesired mutagenesis at some off-target sites because this sgRNA structure has higher sensitivity to mismatches [123].

Another problem is the safe and efficient delivery of genome editing tools to the desired site. There are several problems in delivering these tools in the form of a plasmid or RNA-protein complex (RNP). Nonetheless, there are several systems that have been devised to deliver them. In particular, many studies have been conducted to deliver genome editing tools to in vitro or in vivo models through viral delivery systems such as adenovirus, AAV, and lentivirus [41,43,105]. Viral delivery systems have high delivery efficiency, some of which have been approved for clinical uses [124]. In addition to a viral delivery system, delivery using a non-viral delivery system has also been studied. Several groups have obtained high efficiency using PEGylated liposomes for the in vivo delivery of genome editing tools [39,40].

With the advancement of genome editing technology, ethical issues are also becoming major concerns [125]. The development of a treatment for viral diseases based on genome editing technology targets the viral genome, as well as the human genome. When targeting the human genome, there are cases where research is conducted based on somatic cells. However, some stem cell-based studies are controversial because of the potential for germline editing. Therefore, although genome editing technology has made great progress, there are still many hurdles to overcome before this technology can be applied in clinical practice; efforts are being made to overcome these challenges.

## Figures and Tables

**Table 1:** Comparison between genome editing technologies.

	ZFN	TALEN	CRISPR/Cas
Recognition and binding domains	Zinc finger protein	TAL effector protein	Guide RNA
Genome cleavage domain	Fok1	Fok1	Cas9, Cas13, etc.
Range of binding site	18~36 bp	30~40 bp	20~22 bp
Specifications of binding site	5’-GNGNGNNGNN-3’ type sequences including G base	Sequences starting with the 5’-T base and ending with the A-3’ base	A PAM sequences, such as 5’-NGG-3’, is required immediately after the recognition sequences.
Advantages and Disadvantages	− Small protein size of 1 kb. − Low specificity, complex design, and manufacture of proteins.	− High specificity. − The large protein size makes it difficult to deliver and complicates manufacturing and design processes.	− Target selection is free and easy, and multiple genes can be targeted at once. There is a high probability that off-target effects will occur.

**Table 2:** Summary data of antiviral genome editing technologies against virus types classified based on the Baltimore classification and used in this review.

Group	Virus	Editing Tools	Target	Applications	References
Group I	HPV	TALEN	E7	Effectively downregulated HPV E7 and induced cell death by necrosis.	[32]
		CRISPR/Cas9	E7	Induced apoptosis and growth inhibition in HPV positive cells.	[33]
				Significant inhibition of HPV-induced cancerous activity, both in vitro and in vivo.	[34]
				Decreased E6 expression with activation of the pRb/p21 pathway; this can trigger cellular senescence.	[35]
				Blocking the activity of E7 through targeting the E7 CKII phospho-acceptor site.	[36]
				Improved editing efficiency through co-transfection with dsODN.	[37]
			E6/E7	Significantly upregulated the P53/RB proteins and inhibited the proliferation and motility of HeLa cells.	[38]
				Effectively induced cell death by apoptosis in vivo.	[39,40]
				Apoptosis was induced as the expression level of E6 decreased and the expression level of p53 increased, in vitro and in vivo.	[41]
				Effectively inhibited proliferation of HPV16-positive cervical cancer cells.	[42]
				Effectively targeted the HPV E6 gene and significantly increased the level of p53 protein in HeLa cells.	[43]
			SAMHD1	Controlled HPV-16 induced cell proliferation and viral replication.	[45]
	HSV	CRISPR/Cas9	ICP0	Reduced HSV-1 infectivity in permissive human cell culture models.	[47]
			Multiple genes	Successfully limited productive and latent infections of HSV-1.	[48]
			UL52 and UL29	Completely suppressed HSV-1 infection in vero cells.	[49]
			ICP0 and ICP4	Effectively inhibited the proliferation of HSV-1 without affecting cell viability.	[50]
			gD	Significant reduction in HSV-1 infection.	[51]
			NECTIN-1	HSV infection rate was dramatically decreased in HCECs.	[52]
	EBV	CRISPR/Cas9	Genome	Targeted editing was efficiently achieved in several human epithelial cell lines.	[55]
			LMP1	LMP1-mediated promotion of NPC cell growth was effectively blocked.	[58]
Group IV	HCV	CRISPR/Cas9	CLDN1	Engineered cell line was resistant to HCV infection.	[61]
		CRISPR/Cas13	IRES	Significant inhibition of HCV replication, as well as translation in huh-7.5 cells.	[62]
	ZIKV	CRISPR/Cas9	ADAR1	Significantly reduced ZIKV RNA synthesis in human cell lines.	[66]
			ANKS4B	Viral replication levels were significantly enhanced and showed inhibition of autophagy in ANKS4B-KO cells.	[68]
	CoV	CRISPR/Cas13	Conserved viral region	Designed and screened crRNAs; only six crRNAs can target more than 90% of all coronaviruses.	[72]
			Replicase and nucleocapsid	Selected crRNAs and Cas13a delivery reduced SARS-CoV-2 replication and reduced symptoms in hamsters.	[73]
			Genome	RNA-guided transcript degradation, both in vitro and in eukaryotic cells.	[74]
Group V	IV	CRISPR/Cas13	Conserved viral region	Effectively reduced H1N1 IAV load in respiratory epithelial cells.	[72]
			PB1 and PB2	Reduce viral RNA levels efficiently in cell culture and mice.	[73]
Group VI	HIV	ZFN	CCR5	Stable and heritable protection against HIV-1 infection in vitro and in vivo.	[83]
				HIV RNA became undetectable in one of four patients who could be evaluated, and blood level of HIV DNA decreased in most patients.	[84,85]
				Showed resistance to HIV-1 infection in vitro and in vivo.	[86]
			CXCR4	Lower viral levels in contrast to mice engrafted with unmodified CD4+ T cells.	[87]
			Proviral DNA	The frequency of proviral DNA excision was 45.9% in infected human cell lines.	[88,89]
		TALEN	CCR5	Applied TALENs directly to the primary CD4+ T cells and CD34+ HSCs and helped to create an immune system resistant to HIV-1 infection.	[90]
				Engineered cells were resistant to HIV infection.	[91]
				Protection in a dose-dependent manner is observed in the edited T cells.	[92]
			Proviral DNA	More than 80% of DNA was successfully removed from the T cell lines.	[93]
				TALEN cleaved proviral DNA in vitro and the full-length integrated proviral DNA genome in living cells.	[94]
		CRISPR/Cas9	CCR5	Effectively disrupted the CCR5 gene, and theses cells are resistant to HIV-1 infection.	[95]
				Significant reduction in virus titration and enrichment of human CD4+ T cells.	[96]
				CCR5-disrupted CD4+ T cells showed increased resistance against HIV-1 infection.	[97]
				Edited cells resisted R5-tropic HIV-1 infection.	[98]
			CXCR4	Efficiently induced editing of the CXCR4 gene in human CD4+ cell lines and made these cell lines resistant to HIV-1 infection.	[99]
				Efficiently induced the CXCR4 disruption in an HIV-1 reporter cell line.	[100]
				CXCR4-tropic HIV-1 infections were significantly reduced in CXCR4- modified cells.	[101]
				Efficiently modify both genes at each cell line and showed resistance to HIV-1 infection.	[102]
			Proviral DNA	Completely excised a 9709-bp fragment of integrated proviral DNA, and CRISPR/Cas9 expressing cells prevented HIV-1 infection.	[103]
				HIV-1 gene expression and virus production were significantly diminished.	[104]
				Significantly diminished HIV-1 replication in infected primary CD4+ T-cell cultures and drastically reduced viral load in ex vivo culture of CD4+ T cells.	[105]
				Showed inhibition of viral replication in infected T cell cultures.	[106]
				5′ LTR-driven HIV-1 transcription was significantly reduced in three HIV-1 reporter cell lines.	[107]
				Combinations of two antiviral gRNAs delayed viral escape, and identified two gRNA combinations that durably block virus replication.	[108]
				Completely abrogated viral replication and prevented viral escape in cell culture.	[109]
				Excised HIV-1 proviral DNA in several mouse models.	[110]
				With a combination of CRISPR-Cas9 and RNAi antivirals, observed additive inhibition.	[111]
		CRISPR/Cas13	HIV-1 genome	Efficiently inhibited HIV replication in cell line models.	[112]
Group Ⅶ	HBV	CRIPSR/Cas9	cccDNA	CRISPR/Cas9 direct cleavage reduced viral production in cell lines and reduced cccDNA and HBV protein in a mouse model.	[113]
				Effectively inhibited HBV DNA production in in vitro models of both chronic and de novo HBV infection.	[114]
				Inhibited the replication of HBV, and the viral DNA was significantly reduced in vitro and in vivo.	[115]
				Significantly improved the survival of human hepatocytes and showed a trend toward decreasing total liver HBV DNA and cccDNA.	[116]
				Showed reduced level of HBV infection in cell culture system.	[117]
				Cas9-nickase expression with two sgRNAs cleaved the target HBV genome and suppressed the viral-protein expression and HBV replication in vitro and in vivo.	[118]
				Cas9-BE with certain gRNAs effectively base-edited polymerase and surface genes and reduced HBV gene expression in cells.	[119]

## Data Availability

Not applicable.
